# Molecular detection of tick-borne bacteria and protozoa in cervids and wild boars from Portugal

**DOI:** 10.1186/s13071-016-1535-0

**Published:** 2016-05-10

**Authors:** André Pereira, Ricardo Parreira, Mónica Nunes, Afonso Casadinho, Maria Luísa Vieira, Lenea Campino, Carla Maia

**Affiliations:** Faculty of Veterinary Medicine, Universidade Lusófona de Humanidades e Tecnologias, Lisbon, Portugal; Global Health and Tropical Medicine (GHTM), Instituto de Higiene e Medicina Tropical (IHMT), Universidade Nova de Lisboa (UNL), Lisbon, Portugal; Medical Microbiology Unit, IHMT, UNL, Lisbon, Portugal; Medical Parasitology Unit, IHMT-UNL, Lisbon, Portugal; Department of Biomdical Scienecs and Medicine, Universidade do Algarve, Lisbon, Portugal

**Keywords:** *Anaplasma* spp., Fallow deer, PCR, Portugal, Red deer, *Theileria* spp., Tick-borne pathogens, Wild boar

## Abstract

**Background:**

Wildlife can act as reservoir of different tick-borne pathogens, such as bacteria, parasites and viruses. The aim of the present study was to assess the presence of tick-borne bacteria and protozoa with veterinary and zoonotic importance in cervids and wild boars from the Centre and South of Portugal.

**Methods:**

One hundred and forty one blood samples from free-ranging ungulates including 73 red deer (*Cervus elaphus*), 65 wild boars (*Sus scrofa*) and three fallow deer (*Dama dama*) were tested for the presence of *Anaplasma marginale*/*A. ovis*, *A. phagocytophilum*, *Anaplasma*/*Ehrlichia* spp., *Babesia*/*Theileria* spp., *Borrelia burgdorferi* (*sensu lato*) (*s.l.*), and *Rickettsia* spp. DNA by PCR.

**Results:**

*Anaplasma* spp. DNA was detected in 33 (43.4 %) cervids (31 red deer and two fallow deer) and in two (3.1 %) wild boars while *Theileria* spp. were found in 34 (44.7 %) cervids (32 red deer and two fallow deer) and in three (4.6 %) wild boar blood samples. Sequence analysis of *msp4* sequences identified *A. marginale*, *A. ovis*, while the analysis of *rDNA* sequence data disclosed the presence of *A. platys* and *A. phagocytophilum* and *T. capreoli* and *Theileria* sp. OT3. *Anaplasma* spp./*Theileria* spp. mixed infections were found in 17 cervids (22.4 %) and in two wild boars (3.1 %). All samples were negative for *Babesia* sp., *B. burgdorferi* (*s.l*.), *Ehrlichia* sp. or *Rickettsia* sp.

**Conclusions:**

This is the first detection of *Anaplasma marginale*, *A. ovis*, *A. phagocytophilum, A. platys, Theileria capreoli* and *Theileria* sp. OT3 in cervids and wild boars from Portugal. Further studies concerning the potential pathogenicity of the different species of  *Anaplasma* and *Theileria* infecting wild ungulates, the identification of their vector range, and their putative infectivity to domestic livestock and humans should be undertaken.

## Background

Wildlife can harbor a high density of ticks that can transmit several pathogens, such as bacteria, parasites and viruses. In addition to their veterinary importance, many of these tick-borne pathogens can also affect the human population as a result of their zoonotic potential. Therefore, the management of such situation calls for a One Health approach, including the increased awareness for their presence especially in sylvatic environments and areas associated with animal husbandry among veterinarians, physicians and general public [1].

Piroplasmoses in cattle is caused by tick-borne protozoan parasites comprising several *Theileria* and *Babesia* species. These diseases are a serious health problem, being responsible for important economic losses to the cattle industry. In Europe, infections with different *Theileria* spp. [*Theileria* sp. OT3, *T. capreoli* (formerly *Theileria* sp. 3185/02)*, Theileria* sp. ZS OT4, *T. ovis*] and *Babesia* spp. [including*,* among others, *B. bigemina*, *B. capreoli*, *B. divergens* and *B. venatorum* (formerly *Babesia* sp. EU1)] have been reported in cervids. These include fallow deer (*Dama dama*)*,* red deer (*Cervus elaphus*) and roe deer (*Capreolus capreolus*) [2–5], while *Theileria* sp. and *B. bigemina* were detected in wild boars (*Sus scrofa*) [5]. In Portugal, the main pathogenic piroplasm species reported in cattle is *Theileria annulata*, although others, including *T. buffeli* and *T. orientalis*, considered as being moderately pathogenic or benign, are also present [6, 7]. In addition, several pathogenic species of *Babesia* (*B. bovis*, *B. divergens* and *B. bigemina*) have also been reported in cattle from central and southern Portugal [6, 7]. Further, human babesiosis caused by *B. divergens*, *B. microti* or *B. venatorum* have been reported in several European countries [8], including one fatal case due to *B. divergens* in Portugal [9].

Anaplasmoses, caused by bacteria of the genus *Anaplasma*, known for a long time in veterinary medicine, are also considered as emerging human diseases, and are frequently associated with infection with *Anaplasma phagocytophilum* [10]. This bacterium, which is the causative agent of tick-borne fever, a disease of important negative economic impact to European animal husbandry (involving domestic ruminants), also causes human granulocytic anaplasmosis. Wild ruminants are one of its main reservoirs [11] while the role of wild boars in its natural cycle is still contradictory [12]. Other *Anaplasma* spp. such as *A. marginale* and *A. ovis* have also been detected in European cervids [13]. In Portugal, antibodies reactive to *A. phagocytophilum* antigens were detected in humans and other mammals [14], while *A. marginale* and *A. ovis* were detected in cattle [15] and in sheep [16], respectively.

Among the diseases caused by tick-borne pathogens, Lyme borreliosis caused by spirochetes of the *Borrelia burgdorferi* (*sensu lato*) (*s.l.*) complex is currently the most common tick-borne disease in Europe [17]. In Portugal, its notification in humans is mandatory, but the disease is clearly underdiagnosed and underreported [17]. Wild large vertebrates seem to be frequently exposed to these bacteria, as indicated by the detection of either *Borrelia*-specific antibodies in these animals or *Borrelia* DNA in engorged ticks collected from them [18, 19]. Finally, several tick-borne *Rickettsia* spp. associated with human infections such as *Rickettsia conorii, R. slovaca* and *R. raoultii* have also been described in several European countries, including Portugal [20]. *Rickettsia* spp. (e.g. *R. helvetica*, *R. slovaca*) DNA has previously been detected in the peripheral blood [21] or in ticks removed from cervids and wild boars [4, 22], but the role these wild mammals play in the natural maintenance of these bacteria has not yet been clarified.

No information about tick-borne pathogens circulating in wild ungulates from Portugal is available, with the single exception of the recent detection of *Borrelia burgdorferi* (*s.l*.) in wild boars from northern Portugal [17]. Thus, the aim of the present study was to assess the presence of tick-transmitted bacteria and protozoa with veterinary and zoonotic importance in cervids and wild boars from the Centre and South of the country.

## Methods

### Animals and samples

During the hunting seasons, from December 2013 to March 2015, a total of 141 free-ranging ungulates including 73 red deer (*Cervus elaphus*), 65 wild boars (*Sus scrofa*) and 3 fallow deer (*Dama dama*) from both sexes were sampled in the districts of Castelo Branco (*n* = 31), Portalegre (*n* = 16), Lisboa (*n* = 19), Évora (*n* = 15) and Beja (*n* = 60). Animals were classified in two age categories: young (1–3 years) and adults (> 3 years). Blood samples were collected from each animal by cardiac or thoracic punctures in EDTA tubes and stored at -20 °C until DNA extraction.

### Ethical approval

This study was ethically approved by the board of the Faculty of Veterinary Medicine (ULHT).

### PCR amplification

A commercial kit (PCR-template Preparation kit, Roche Diagnostics GmbH, Germany) was used to extract DNA from the collected blood samples, following the manufacturer’s instructions.

In order to avoid false negative results due to PCR inhibition, and so as to validate the efficiency of the DNA extraction, the modified vertebrate-universal *cyt-b* specific primers (cytB1-F and cytB2-R) were used to amplify a 350 bp segment of the host mitochondrial *cytochrome b* gene (*cyt-b*) [23]. PCR amplifications were performed in a 25 μl final volume containing 12.5 μl of NZYTaq 2x Green Master Mix (Nyztech, Portugal), 1 μl of each primer (10 pmol) and 2 μl of template DNA.

Detection of *Anaplasma*/*Ehrlichia* spp., *A. marginale*/*A. ovis*, *A. phagocytophilum*, *Babesia/Theileria* spp., *B. burgdorferi* (*s.l.*) and *Rickettsia* spp. DNA in blood samples was assessed by PCR, according to previously described protocols (Table 1).

**Table 1:** Sequences of the oligonucleotide primers used

Pathogen	Target gene		Oligonucleotide sequences (5′-3′)		Amplicon size (bp)	Reference
			Forward	Reverse		
*Anaplasma* spp./*Ehrlichia* spp.	*16S rRNA*		GGTACCYACAGAAGAAGTCC	TAGCACTCATCGTTTACAGC	345	[38]
	*groEL*		ACTGATGGTATGCARTTTGAYCG	TCTTTRCGTTCYTTMACYTCAACTTC	600	[39]
*Anaplasma marginale/A. centrale/A. ovis*	*msp4*		GGGAGCTCCTATGAATTACAGAGAATTGTTTAC	CCGGATCCTTAGCTGAACAGGAATCTTGC	851	[40]
*Anaplasma phagocytophilum*	*msp4*		ATGAATTACAGAGAATTGCTTGTAGG	TTAATTGAAAGCAAATCTTGCTCCTATG	849	[13]
*Babesia* spp./*Theileria* spp.	*18S rRNA*		AATACCCAATCCTGACACAGGG	TTAAATACGAATGCCCCCAAC	400	[38]
*Borrelia burgdorferi* (*sensu lato*)	*ITS 5S-23S rRNA*	Outer primmers	ACCATAGACTCTTATTACTTTGAC	TAAGCTGACTAATACTAATTACCC	380	[41]
		Inner primers	ACCATAGACTCTTATTACTTTGACCA	GAGAGTAGGTTATTGCCAGGG	225	
	*flaB*	Outer primmers	TGGTATGGGAGTTTCTGG	TAAGCTGACTAATACTAATTACCC	774	[42]
		Inner primers	CAGACAACAGAGGGAAAT	TCAAGTCTATTTTGGAAAGCACC	604	
*Rickettsia* spp.	*gtlA*		GGGGGCCTGCTCACGGCGG	ATTGCAAAAAGTACAGTGAACA	381	[43]

PCR amplifications were performed in a final volume of 25 μl using NZYTaq 2× Green Master Mix, 3 μl of the prepared DNA extracts and 10 pmol of each primer. In all amplifications, positive (containing genomic DNA of the targeted microrganism) and negative (without DNA) controls, were included. PCR amplifications were carried out in a Thermo Electron Corporation® Px2 Termal Cycler (VWR, USA) and the obtained PCR products visualized under UV illumination after electrophoresis on 1.5 % agarose gels stained with Greensafe premium® (Nzytech, Portugal) using a 100 bp DNA ladder as a molecular-weight size marker (Nzytech, Portugal).

PCR products were purified from agarose gel slices with NZYGelpure® (Nzytech, Portugal) according to the manufacturer’s instructions. Purified products were sent to LIGHTrun™ Sequencing Service (GATC-biotech, Germany) for direct sequencing of the obtained amplicons by Sanger’s method with the same primers used for DNA amplification.

### DNA sequence analyses

Species identity of the obtained sequences was assessed on the basis of the closest BLASTn match (identity ≥ 98 % using the MegaBLAST and a query cover no smaller than 96 %) with homologous sequences deposited in the GenBank database. The sequences obtained in the course of this work were deposited at DNA Data Bank of Japan (DDBJ) (http://www.DDBJ.nig.ac.jp).

Phylogenetic relationships were inferred from nucleotide sequence alignments produced with the MAFFT multiple alignment program using a combination of the G-INS-i alignment option [24]. Phylogenetic tree construction was carried out using a Maximum Likelihood (ML) approach, using the Kimura’s 2-P (K2P) evolutionary model, and assuming a Γ distributed substitution rates among sites, as indicated by Mega6 [25] on the basis of the Akaike information criterion. Alternatively, an empirically defined model (GTR + Γ + I) was also used. The topological robustness of the obtained trees was assessed by bootstrapping, using 1000 resampling of the original alignment data. The final trees were manipulated for display using FigTree v.1.2.2. (available at http://tree.bio.ed.ac.uk/software/figtree/).

### Statistical analysis

Percentages of positive samples for *Anaplasma* spp. and *Theileria* spp. regarding the independent variables and categories were compared by the Chi-square or Fisher’s exact tests. A *P*-value ≤ 0.05 was considered as statistically significant. Exact binomial 95 % confidence intervals (CI) were defined for the proportions. Analyses were performed with Epi Info™ 7.1.5.2 software for Centers for Disease and Prevention.

## Results

A 350 bp fragment of the host mitochondrial *cyt-b* gene was amplified in all DNA blood samples.

*Anaplasma* spp. DNA was detected in 33 (43.4 % CI: 32.1–55.3 %) cervids (31 red deer and two fallow deer) and in two (3.1 % CI: 0.4–10.7 %) wild boars using a set of general primers that target *16S rDNA*. Seventeen sequences obtained from red deer (accession numbers: LC126854, LC126858-9, LC126863-5, LC126867-9, LC126871, LC126873, LC126875, LC126878, LC126879 and LC126881-3) and two from wild boars (accession numbers: LC126885-6) showed 99–100 % identity with *A. platys* previously described in dogs from Portugal (LC018182-3; [26]), Argentina (JX261979; [27]) and in a goat from Cyprus (EU090182; [28]). Further, eight sequences obtained from red deer (LC126855-6, LC126860-2, LC126870, LC126876 and LC126884) showed 99 % identity with *A. phagocytophilum* described in Swedish moose (KC800983; [29]) and in black-striped field mice (*Apodemus agrarius*) in South Korea (KR611719). Six sequences isolated from red deer (LC126857, LC126866, LC126872, LC126874 and LC126880) and two isolated from fallow deer (LC126852-3) showed 100 % identity with *A. marginale* (KC335218, KC335223) described in cows and with *A. ovis* described in sheep (KC335231) and goats (KC335225) from Italy [30] as well as with *A. centrale* described in goats from China (KP062964, KP062966; [31]).

**Fig. 1:**
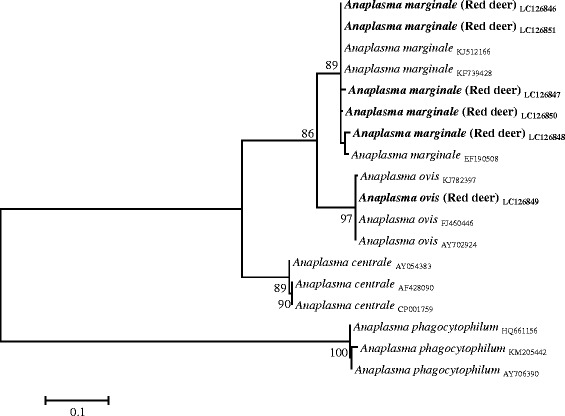
Phylogenetic tree of *Anaplasma spp.* based on the analysis of *msp4* sequences

Sequencing of the *msp4* gene amplified from the samples where the presence of *A. centrale/A.marginale/A. ovis* DNA had been detected with the *16S rDNA* primers, confirmed the presence of *A. marginale* in five (6.6 % CI: 2.2–14.7 %) red deer from the Castelo Branco, Portalegre and Beja districts and *A. ovis* in one (1.3 % CI: 0–7.1 %) red deer from Beja. Attempts to amplify *msp4* sequences from the two fallow deer failed. The obtained *msp4* sequence data, along with related sequences obtained from Genbank, were subjected to phylogenetic analyses (Fig. 1).

**Fig. 2:**
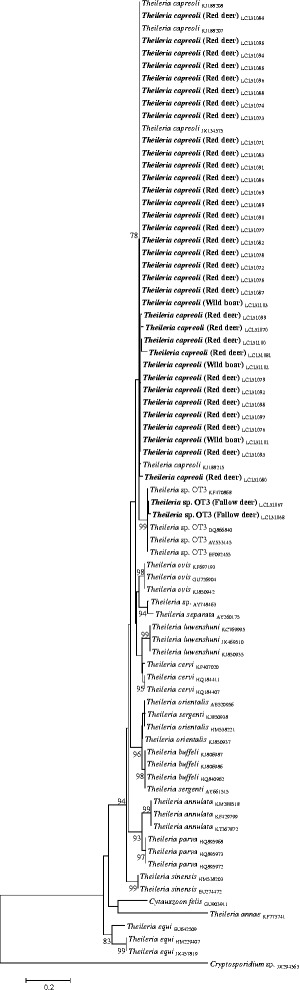
Phylogenetic tree of *Theileria* spp. based on *18S rRNA* gene sequences

PCR reactions prepared using either *groEL* or *A. phagocytophilum* species-specific primers revealed reproducibly negative amplification results. On the contrary, *Theileria* spp. were found in 34 (44.7 % CI: 33.3–56.6 %) cervids (32 red deer and two fallow deer) and in three (4.6 % CI: 1.0–12.9 %) wild boar samples, using primers targeting the *18S rRNA* gene. Blast analysis showed that the sequences obtained from red deer (accession numbers: LC131069-100) and wild boars (LC131101-3) presented 98–99 % identity to *T. capreoli* (KJ188207-8) described in Sika deer from China [32] while the two sequences obtained from fallow deer (LC131067-8) showed a high identity (98–99 %) to the *Theileria* sp. OT3 (Genbank: KF470868) described in sheep from China [33]. The phylogenetical analysis of the obtained *18S rDNA* sequences along with the related sequences from GenBank corroborated the Blast identification (Fig. 2).

*Anaplasma* spp./*Theileria* spp. co-infections were found in 17 cervids (22.4 % CI: 13.6–33.4 %). Of these, eight red deer were co-infected with *A. platys* and *T. capreoli*, four with *A. marginale* and *T. capreoli*, two with *A. phagocytophilum* and *T. capreoli*, one with *A. ovis* and *T. capreoli*, while two fallow deer were co-infected with *Anaplasma* sp. and *Theileria* sp. OT3. Two wild boars (3.1 % CI: 0.4–10.7 %) were co-infected with *A. platys* and *T. capreoli*.

The frequency of *Anaplasma* infection was significantly higher (*P* = 0.019) in red deer from the Castelo Branco district than from Beja (Table 2). All the blood samples taken from wild boars with positive PCR amplification results to *T. capreoli* or *A. platys* were from the Beja district.

**Table 2:** Prevalence of tick-borne pathogens as detected by PCR in 76 cervids and 65 wild boars from Centre and southern Portugal

Idependent variable/category	*Cervus elaphus & Dama dama*					*Sus scrofa*				
	No. (%)	*Anaplasma* spp.		*Theileria* spp.		No. (%)	*Anaplasma* spp.		*Theileria* spp.	
		% of positive	95 % CI	% of positive	95 % CI		% of positive	95 % CI	% of positive	95 % CI
District	76	ND*		ND*		65	ND*		ND*	
Castelo Branco	27 (35.5)	59.3^a^	38.8–77.6	44.4	25.5–64.7	4 (6.2)	0	0–60.2	0.0	0.0–60.2
Portalegre	1 (1.3)	100	2.5–100	100	2.5–100	15 (23.1)	0	0–21.8	0.0	0.0–21.8
Lisboa	3 (3.9)	66.7	9.4–99.2	66.7	9.4–99.2	16 (24.6)	0	0–20.6	0.0	0.0–20.6
Évora	–	–	–	–	–	15 (23.1)	0	0–21.8	0.0	0.0–21.8
Beja	45 (59.2)	31.1^a^	18.2–46.7	42.2	27.7–57.9	15 (23.1)	13.3	1.66–40.5	20.0	4.3–48.1
Age	61	*P* = 0.309		*P* = 0.607^c^		62	*P* = 1		*P* = 0.545	
Adult	50 (82.0)	40.0	26.4–54.8	46.0	31.8–60.7	42 (67.7)	4.8	0.6–16.2	7.1	1.5–19.5
Young	11 (18.0)	18.2	2.3–51.8	54.5	23.4–83.3	20 (32.3)	0	0–16.8	0.0	0.0–16.8
Gender	69	*P* = 0.618^b^		*P* = 0.025^d^		63	*P* = 0.493		*P* = 1	
Female	37 (53.6)	37.8	22.5–55.2	32.4	18.0–49.8	45 (71.4)	2.2	0.1–11.8	4.4	0.5–15.2
Male	32 (46.4)	43.8	26.4–62.3	59.4	40.7–76.3	18 (28.6)	5.6	0.1–27.3	5.6	0.1–27.3
Total	76	43.4	32.1–55.3	44.7	33.3–56.6	65	3.1	0.4–10.7	4.6	1.0–12.9

^a^ *χ* ^2^ = 5.50, *df* = 1, *P* = 0.019

^b^ *χ* ^2^ = 0.25, *df* = 1

^c^ *χ* ^2^ = 0.26, *df* = 1

^d^ *χ* ^2^ = 5.03, *df* = 1

ND* Statistically significant difference(s) not confirmed after pairwise comparisons

None of the samples analysed revealed the presence of *Babesia* sp., *B. burgdorferi* (*s.l.*), *Ehrlichia* sp. or *Rickettsia* sp.

## Discussion

Concern about the role of wildlife in the natural maintenance transmission of tick-borne pathogens is increasing, especially in areas where free-ranging animals regularly interact with domestic livestock and humans [5, 34].

This study, which aimed at the detection of tick-borne bacteria and protozoa of veterinary and zoonotic importance in cervids and wild boars, disclosed, to our knowledge, the first evidence for the circulation of *Anaplasma* spp. and *Theileria* spp. among red deer, fallow deer and wild boars in central/southern Portugal. In this study, *Anaplasma* spp. infections were detected in the three wild ungulate species analysed as revealed by the amplification of *16S rDNA* sequences using genus-specific primers. *Anaplasma platys* causes canine cyclic thrombocytopenia and is presumably transmitted by ticks of the *Rhipicephalus sanguineus* group. As *A. platys* DNA has previously been reported in dogs [26], ticks [35] and red foxes [36] from Portugal, its detection in the red deer and wild boars sampled herein indicates that these animals are also exposed to the bacterium. However, the ability of *A. platys* to cause disease in these animals has not been established yet.

As wild cervids are considered one of the main reservoirs of *A. phagocytophilum* [11], the detection of this bacterium in eight red deer blood samples using *16S rDNA* primers it is not entirely surprising, especially when it is known that the pathogen is circulating in different Portuguese vertebrate hosts, as well as in *Ixodes ricinus,* its most frequently associated vector [14]. However, the absence of detection of *msp4* specific sequences may indicate the circulation of divergent *A. phagocytophilum* variants among Portuguese red deer different from the ones previously reported [37]. In any case, this issue deserves future clarification. Furthermore, the presence of *A. ovis* and *A. marginale* in red deer was confirmed by *msp4* phylogenetic analysis, confirming the susceptibility of this cervid to the agents responsible for bovine and ovine anaplasmoses [13]. Both pathogens have been reported in cattle from the Alentejo region (which includes the Évora and Beja districts [15]), and in sheep raised throughout the country [16].

The occurrence of *Theileria* spp. infections in European cervids is well documented [2–5]. In the present study, and for the first time in Portugal, *T. capreoli* and *Theileria* sp. OT3 *msp4* sequences were amplified from red deer and fallow deer samples, respectively, corroborating previous data from northern Spain [3]. The overall prevalence of *Babesia* spp. and *Theileria* spp. infections previously reported in cattle from the central and southern regions of Portugal ranged from 23.1 % [7] to 74.7 % [6], respectively, with *T. annulata* and *T. buffeli* being the most commonly detected species and *B. bigemina*, *B. bovis* and *B. divergens* being the  least frequently found. As none of the *Theileria* and *Babesia* species known to circulate in the Portuguese cattle were detected in the present study, it seems that the tested wild ungulate species might not play a significant role in their transmission, at least in the regions where samples were collected. Furthermore, although deer have been previously appointed as the source for ovine infection with *Theileria* sp. OT3 [3], no data is yet available regarding the circulation of piroplasmids in small ruminants from Portugal.

Despite the fact that *B. burgdorferi* (*s.l*.) and *Rickettsia* spp. have already been detected in ticks and/or in the blood collected from cervids and wild boars [4, 17, 19, 22], their presence was not revealed in any of the samples analysed in the present study. This observation supports the hypothesis that wild ungulates, at least in the studied areas, are not pivotal players in the natural maintenance cycles of these bacteria, as previously reported [4, 21].

As large wildlife are important to maintain tick populations, and since ticks may become infected with several pathogens during their life cycle, the detection of *Anaplasma* spp./*Theileria* spp. co-infections in the present study is not surprising, and falls in line with previously published observations in wild ungulates [4]. The interaction of different pathogens within the vertebrate host might lead to increased susceptibility to other infections as well as a modification of the pathogenesis of each microorganism with profound consequences for disease management programs and wildlife conservation [4].

## Conclusions

The present study provides information regarding the presence of *Anaplasma marginale*, *A. ovis*, *A. phagocytophilum, A. platys, Theileria capreoli* and *Theileria* sp. OT3 in cervids and wild boars from central and southern Portugal. Further studies concerning the potential pathogenicity of the different *Anaplasma* and *Theileria* species infecting wild ungulates, the identification of their vector range, and their infectivity to domestic livestock and humans should be undertaken.
